# Identification of M0 macrophage associated lipid metabolism genes for prognostic and immunotherapeutic response prediction in hepatocellular carcinoma

**DOI:** 10.1007/s12672-025-02620-1

**Published:** 2025-05-16

**Authors:** Huanjie Zhou, Ming Lao, Zhengui Liang, Huiliu Zhao, Ying Wang, Qiongqing Huang, Chao Ou

**Affiliations:** Department of Clinical Laboratory, Guangxi Medical University Cancer Hospital, Guangxi Zhuang Autonomous Region, Nanning, 530021 People’s Republic of China

**Keywords:** Lipid metabolism genes, M0 macrophages, Metabolic reprogramming, HCC

## Abstract

**Purpose:**

Liver cancer prognosis is associated with M0 macrophages and lipid metabolism reprogramming; however, the prognostic role of M0 macrophage-related lipid metabolism genes in hepatocellular carcinoma (HCC) remains unclear.

**Methods:**

We identified 153 lipid metabolism genes associated with M0 macrophage infiltration in HCC from The Cancer Genome Atlas (TCGA) and the Molecular Signatures Database (MSigDB). Prognostic genes were selected, and a model was constructed using least absolute shrinkage and selection operator (LASSO) and Cox regression analyses. The model was validated using the International Cancer Genome Consortium (ICGC) database. We assessed the expression levels of prognostic genes by quantitative real-time polymerase chain reaction (qRT‒PCR).

**Results:**

A prognostic model was developed based on five characteristic genes. Receiver operating characteristic curve analysis demonstrated that the model had good accuracy, with area under the curve values of 0.796, 0.732, and 0.728 for predicting survival at 1, 3, and 5 years, respectively. The high-risk group exhibited increased sensitivity to common chemotherapy drugs, including sorafenib, dasatinib, and 5-fluorouracil, compared with the low-risk group (P < 0.05). Additionally, the high-risk group had significantly more infiltrating M0 macrophages, resting dendritic cells, follicular helper T cells, and regulatory T cells than did the low-risk group (P < 0.05). The qRT‒PCR results confirmed the upregulation of these five characteristic genes in HCC tissues.

**Conclusions:**

M0 macrophage-associated lipid metabolism genes may serve as biomarkers for the prognosis of patients with HCC and as targets for immunotherapy.

**Supplementary Information:**

The online version contains supplementary material available at 10.1007/s12672-025-02620-1.

## Introduction

Hepatocellular carcinoma (HCC) is a prominent factor in cancer-related deaths [1], presenting a considerable challenge to global health systems. The heterogeneity within tumours significantly reduces the effectiveness of existing treatment options, resulting in low five-year survival rates and median survival durations of only 6–10 months [2, 3]. Thus, it is vital to explore effective strategies for assessing patient prognosis and investigating the molecular mechanisms at play.

The tumour microenvironment (TME) of solid tumours is a highly heterogeneous ecosystem that plays a pivotal role in the clinical response to treatment. Invasive tumour-associated macrophages (TAMs) represent crucial immune cell populations within the TME that profoundly influence tumorigenesis and progression [4] and are strongly associated with unfavourable prognoses across various cancer types [5]. Consequently, TAMs have emerged as promising targets for cancer therapy [6]. Within the microenvironment, TAMs can polarize into distinct functional states [7], and M0 macrophages can transition from an inactive state to either the M1 (proinflammatory) or the M2 (anti-inflammatory) phenotype [8]. Several studies have shown that the abundance of M0 macrophages in tumours correlates with patient survival outcomes [9, 10]. These findings highlight the possible tumorigenic role of M0 macrophages.

Dyslipidaemia is considered a driving force behind liver cancer, particularly the modulation of fatty acid and cholesterol metabolism [11]. Numerous studies have consistently demonstrated that the activation of lipid metabolism in tumours results in the accumulation of a plethora of lipid metabolites, thereby fostering tumour progression and contributing to local TME heterogeneity [12] while also influencing TAM metabolism and functional remodelling. Research indicates that long-chain fatty acid (LCFA) metabolism in tumours can regulate the immunosuppressive phenotype of TAMs [13]. Fatty acid oxidation and glutamine metabolism are regulated by activated CD40, which promotes M1 macrophage polarization and enhances macrophage antitumour function [14]. Resting M0 macrophages are typically regarded as precursors for polarized macrophages, with both M1 and M2 phenotypes arising from them. Investigating the impact of lipid metabolism gene expression on M0 macrophage infiltration and activation will help elucidate TAM regulatory pathways in cancer therapy. Therefore, there is an urgent need to screen and characterize lipid metabolism genes associated with M0 macrophages to provide evidence for further understanding the mechanisms of interaction among liver cancer, lipid metabolism, and macrophages.

In this study, we conducted a prognostic analysis of M0 macrophages and identified lipid metabolism genes associated with the abundance of infiltrating M0 macrophages using CIBERSORT analysis. We subsequently developed a prognostic risk model for HCC and validated its effectiveness in an independent cohort. Furthermore, we evaluated the predictive ability of the model for immunotherapy response. Additionally, we preliminarily confirmed the expression of these signature genes in HCC tissues. Our findings contribute to a better understanding of the combined impact of M0 macrophages and lipid metabolism genes on HCC prognosis while providing valuable insights for clinical decision-making and potential therapeutic targets.

## Materials and methods

### Data collection

A total of 344 tumour samples were obtained by downloading the gene expression and clinical data from The Cancer Genome Atlas (TCGA) liver hepatocellular carcinoma (LIHC) cohort using the “TCGAbiolinks” R package. The International Cancer Genome Consortium (ICGC) database (https://dcc.icgc.org/) was used to retrieve the gene expression and clinical data of the ICGC Liver Cancer-RIKEN, JP (LIRI-JP) cohort, and 237 tumour samples were obtained. Data from both cohorts excluded patients whose overall survival (OS) time was less than 30 days or whose clinical data were incomplete. Single-cell RNA sequencing (scRNA-seq) data were obtained from the GSE149614 dataset in the Gene Expression Omnibus (GEO) (accessed January 23, 2024). We obtained a total of 843 lipid metabolism-related genes from the Molecular Signatures Database (MSigDB) (http://www.broad.mit.edu/gsea/msigdb/, accessed November 14, 2023). All datasets complied with the access rules for their respective databases.

### Lipid metabolism genes associated with M0 macrophage infiltration

We used the ‘CIBERSORT’ R package to assess the abundance of infiltrating M0 macrophages in the tumour samples. We constructed a Kaplan–Meier survival curve to illustrate the link between M0 macrophage infiltration and HCC prognosis. We performed Pearson correlation analysis to examine the relationship between M0 macrophage infiltration and log10-transformed HCC gene expression. We retained genes with absolute correlations greater than 0.2 (|correlation|> 0.2) and p values less than 0.05 (P < 0.05). We visualized overlapping lipid metabolism genes using the ‘VennDiagram’ R package. The R packages utilized for these analyses were ‘survival’, ‘survminer’, ‘tidyverse’, ‘CIBERSORT’, ‘ggplot2’, and ‘dplyr’.

### Functional enrichment analysis

Gene Ontology (GO) and Kyoto Encyclopedia of Genes and Genomes (KEGG) functional enrichment analyses were performed to explore specific functional categories of lipid metabolism genes related to M0 macrophages. The packages ‘ClusterProfiler’, ‘org.Hs.eg.db’, ‘stringr’, ‘ggplot2’, and ‘enrichplot’ were used for these analyses.

### Development and validation of prognostic signature genes

We employed least absolute shrinkage and selection operator (LASSO) analysis to identify prognosis-related genes. Univariate Cox regression analysis was subsequently conducted to select variables with p values less than 0.05, and stepwise regression analysis was used to finalize the set of characteristic genes. Multivariate Cox regression analysis was subsequently performed to develop a prognostic model. The risk score calculation formula is as follows: risk score = h0(t) * exp(β1X1 + β2X2 + … + βnXn), where β represents the regression coefficient, X indicates covariate, and h0(t) denotes the baseline risk function. Patients in both the training and validation cohorts were categorized into high-risk and low-risk groups based on the median risk score. Kaplan‒Meier survival curves were plotted to assess differences between these groups, and receiver operating characteristic (ROC) curves and calibration curves were used to evaluate model performance. Forest plots were constructed to illustrate the variable indices from both univariate and multivariate Cox regression analyses. The relationships between risk scores and clinicopathological characteristics were analysed using the chi-square test and Wilcoxon rank-sum test, and a nomogram was created to predict patient OS. The R packages used in these analyses included ‘MASS’, ‘glmnet’, ‘survival’, ‘rms’, ‘survminer’, ‘ggrisk’, ‘forestplot’, and ‘pheatmap’.

### Evaluation of mutant genes and drug sensitivity prediction

TCGA-LIHC mutation data were acquired using the “TCGAbiolinks” R package, and a waterfall plot was created to compare mutation frequencies and distributions between the high- and low-risk score groups. We also analysed the correlations between risk scores and common chemotherapy agents (|correlation|> 0.2, P < 0.05). The “oncoPredict” R package was employed to calculate the half-maximal inhibitory concentration (IC_50_), and a Wilcoxon rank-sum test was conducted to assess differences between groups. The R packages used in these analyses included “maftools”, “oncoPredict”, “ggplot2”, and “ggpubr”.

### Evaluation of immune cell infiltration and the immune microenvironment

Changes in immune cell infiltration between the high- and low-risk score groups were evaluated using the QUANTISEQ, TIMER, MCPCOUNTER, EPIC, CIBERSORT-ABS, CIBERSORT, and XCELL methods. Spearman correlation analysis was performed to assess the relationship between the risk score and the abundance of infiltrating immune cells (|correlation|> 0.2, P < 0.05). Linear correlation graphs were created to demonstrate the associations between tumour necrosis factor (TNF) and human leukocyte antigen (HLA) gene expression and the risk score. The R packages applied for these analyses included “IOBRA”, “Hmisc”, “tidyverse”, and “ggplot2”.

### Analysis of prognostic genes using scRNA-seq

Tumour samples were obtained from the GSE149614 dataset, and the scRNA-seq data were filtered using the following criteria: 500–4,000 unique gene counts, more than 200 genes, and fewer than 5% mitochondrial counts. The"NormalizeData"and"Harmony"methods were employed for batch correction and data integration. Principal component analysis (PCA), cluster analysis, and uniform manifold approximation and projection (UMAP) were subsequently performed for dimensionality reduction and visualization of the integrated dataset. Annotations were assigned to the clusters using the “SingleR” method. The R packages utilized for these analyses included “Seurat”, “Harmony”, “Tidyverse”, “Patchwork”, “SingleR”, and “dplyr”.

### Cell culture

The HepG2 and HCCLM3 cell lines were obtained from Servicebio Biotechnology (Hongshan, Wuhan, China), and the PLC/PRF/5 and MIHA cell lines were acquired from the Shanghai Cell Bank (Xuhui, Shanghai, China). All the cell lines were cultured in Dulbecco's modified Eagle’s medium (DMEM) supplemented with 10% foetal bovine serum (XP BioMed Biotechnology, Fengxian, Shanghai, China), and maintained with 100 μg/ml streptomycin and 100 U/ml penicillin (Solarbio Biotechnology, Tongzhou, Beijing, China) to prevent microbial contamination.

The cultures were maintained in a humidified incubator at 37 °C with an atmosphere of 5% CO_2_, which is critical for optimal cellular growth and viability. The culture medium was refreshed every two days to provide essential nutrients and remove metabolic waste, ensuring a stable environment conducive to cell proliferation. This rigorous cell culture protocol is fundamental for ensuring the reliability and reproducibility of experimental results in hepatocellular carcinoma research.

### RNA extraction and quantitative real-time polymerase chain reaction (qRT‒PCR)

Samples of 49 pairs of HCC and adjacent tissues were obtained. The study was approved by the Ethics Review Committee of Guangxi Medical University Cancer Hospital (LW2024032). Individual consent was waived for this study, and all patient data were anonymized or deidentified. Total RNA was isolated using QIAzol Lysis reagent (QIAGEN, Lot No. 560012414, USA). The RNA was reverse transcribed using a reverse transcription kit (Takara, Cat No. RR047 A, Japan). qRT‒PCR was conducted with a ChamQ Universal SYBR QPCR MasterMix Kit (Vazyme, Lot No. 7E891B4, China) following the manufacturer's instructions. Relative mRNA expression was determined using the 2-ΔΔCt method. All primers were synthesized by Sangon Biotech (Shanghai, China) and are listed in Supplementary File S1.

### Statistics

Statistical analyses were performed using R version 4.2.1 (https://www.r-project.org/). Student's t-test and the Wilcoxon rank-sum test were used to compare the two groups. Kaplan–Meier survival curves and the log-rank test were used to assess differences in OS between the groups. Statistically significant differences were indicated by p values < 0.05.

## Results

### Defining lipid metabolism genes associated with M0 macrophages

**Fig. 1:**
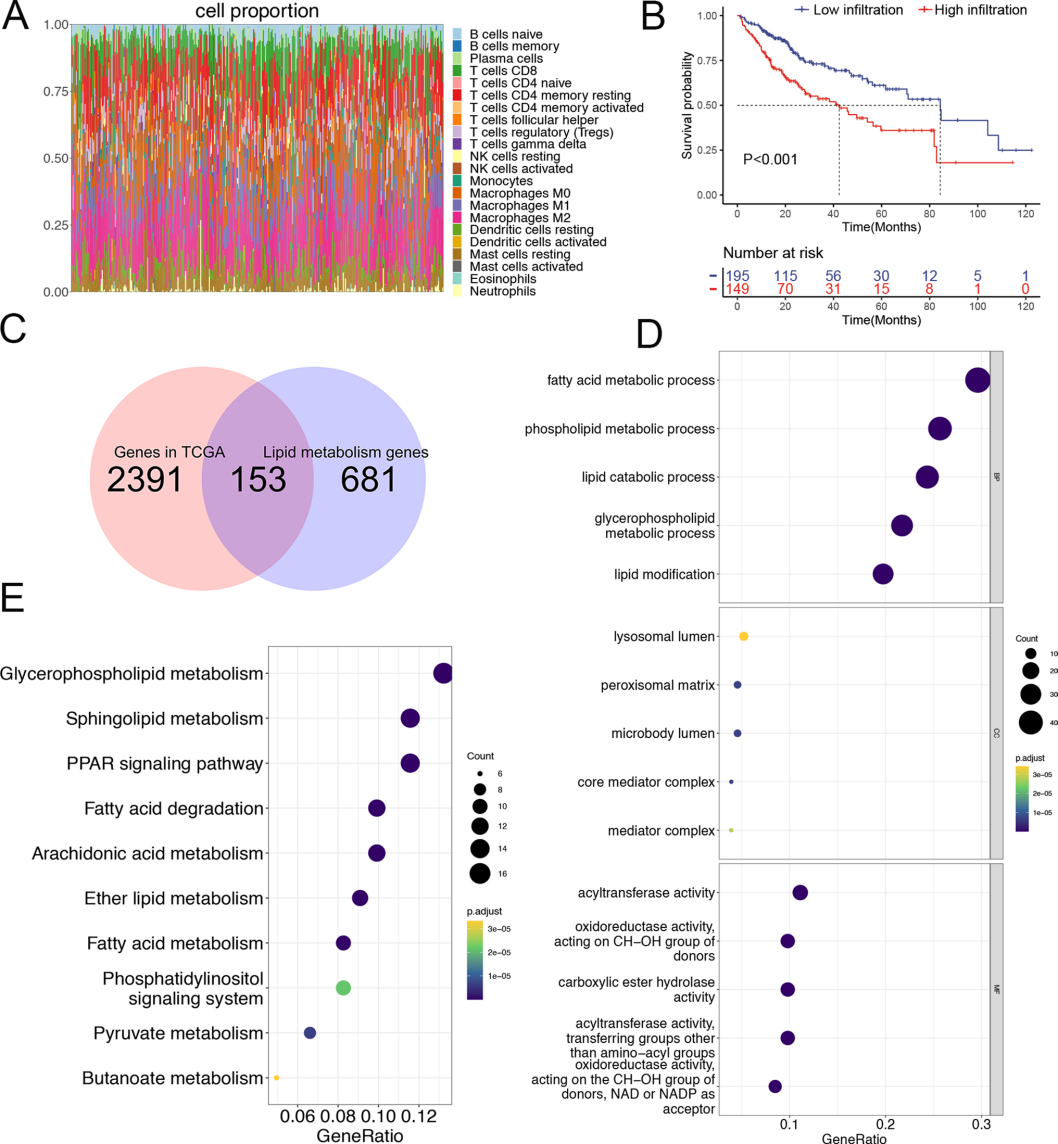
Identification of lipid metabolism-associated genes in M0 macrophages. **A** CIBERSORT analysis illustrating immune cell infiltration in the TCGA-LIHC tumour dataset. **B** Kaplan–Meier survival curve analysis showing differences in OS between the high and low M0 macrophage infiltration groups. **C** Intersection of M0 macrophage-related genes with lipid metabolism-related genes. GO **D** and KEGG **E** enrichment analyses of the 153 lipid metabolism-associated genes in M0 macrophages. *TCGA* The Cancer Genome Atlas, *LIHC* liver hepatocellular carcinoma, *OS* overall survival, *GO* Gene Ontology, *KEGG* Kyoto Encyclopedia of Genes and Genomes

CIBERSORT analysis revealed that M0 macrophage infiltration levels in the TCGA-LIHC dataset were significantly associated with the prognosis of patients with HCC (Fig. 1A). Kaplan–Meier survival curves revealed that patients with high infiltration had a significantly shorter OS than those with low infiltration (Fig. 1B). We then analysed genes correlated with M0 macrophage infiltration (|correlation|> 0.2, P < 0.05), identifying 2,544 genes that overlapped with 834 lipid metabolism-related genes. Ultimately, we identified 153 lipid metabolism-related genes specifically associated with immune infiltration by M0 macrophages (Fig. 1C). Further investigation through GO and KEGG enrichment analyses revealed their potential biological roles. GO analysis (Fig. 1D) indicated significant involvement in fatty acid metabolic processes, phospholipid metabolic processes, and lipid catabolic processes within biological processes (BP). Notably, the enriched cellular components (CC) included lysosomal lumen, peroxisomal matrix, and microbody lumen. The key molecular functions (MF) included acyltransferase activity, oxidoreductase activity, CH-OH group donor activity, and carboxylic ester hydrolase activity. Additionally, KEGG analysis indicated that these genes were enriched primarily in glycerophospholipid metabolism, sphingolipid metabolism, the PPAR signalling pathway, fatty acid degradation, and arachidonic acid metabolism (Fig. 1E).

### Establishment and validation of a prognostic model of characteristic genes

**Fig. 2:**
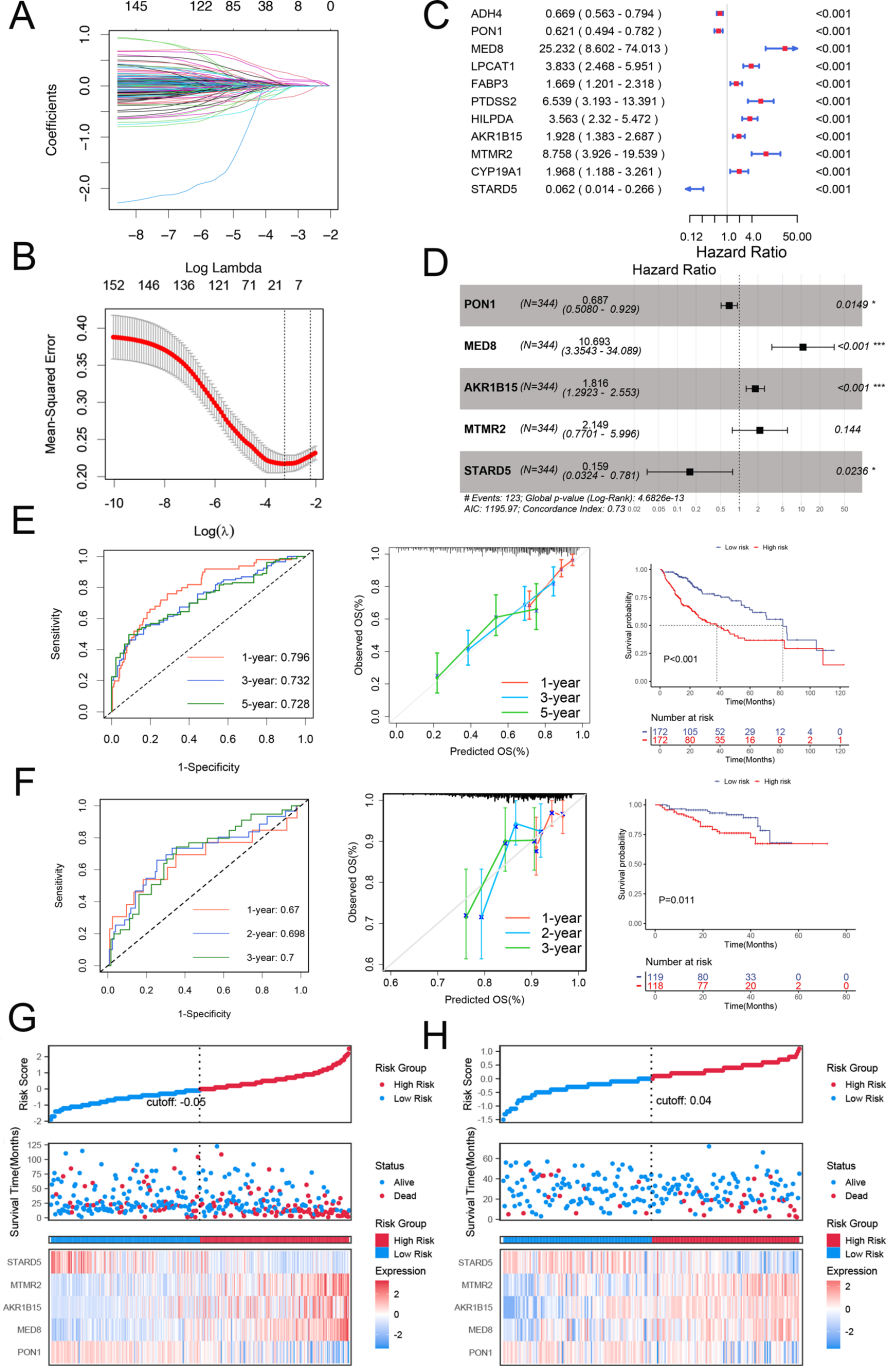
Construction and validation of a prognostic model for characteristic genes. **A** LASSO coefficient profiles. **B** Parameter selection in the LASSO model. **C** Forest plot illustrating eleven indicators from univariate Cox regression analysis (P < 0.05) after LASSO analysis. **D** Forest plot depicting five characteristic genes for Cox regression analysis. ROC curves and calibration curves were used to display the survival probabilities of patients with HCC, whereas the Kaplan–Meier survival curve demonstrated the difference in OS between the high- and low-risk groups in both the training cohort **E** and the validation cohort **F**. The risk plot of the prognostic model displayed the distribution of risk scores and OS outcomes, alongside the expression of the five signature genes in the training cohort **G** and validation cohort **H**. *LASSO* least absolute shrinkage and selection operator, *ROC* receiver operating characteristic, *HCC* hepatocellular carcinoma, *OS* overall survival

Among the 153 identified genes, we developed and validated a prognostic model for HCC. The TCGA-LIHC dataset (344 samples) served as the training cohort, and the ICGC-LIRI-JP dataset (237 samples) served as the validation cohort. Using LASSO analysis (Fig. 2A, B) and univariate Cox regression analyses (P < 0.05), we identified eleven characteristic genes (Fig. 2C). Through backwards selection and multivariate Cox regression analysis, we subsequently confirmed five feature genes associated with M0 macrophage infiltration for model construction: PON1, MED8, AKR1B15, MTMR2, and STARD5 (Fig. 2D). The risk scores were calculated using the following formula: risk score = h0(t) * exp (MED8 * 2.3696–PON1 * 0.3752 + AKR1B15 * 0.5969 + MTMR2 * 0.7649—STARD5 * 1.8381). By applying this formula to each sample in our study cohort, patients were categorized into high- and low-risk score groups based on their median risk scores (Fig. 2G, H). Kaplan–Meier survival curve analysis revealed that patients in the high-risk score group had poor OS (P < 0.05). To assess our model's performance, we computed the ROC curves at different time points; specifically, the area under the curve (AUC) values at 1 year, 3 years, and 5 years were 0.796, 0.732, and 0.728 (Fig. 2E), respectively, indicating the robust accuracy of our model predictions over these time intervals. The calibration curve demonstrated the strong predictive ability of our model. Furthermore, the validation cohort also exhibited excellent predictive ability and broad applicability through risk score modelling and Kaplan–Meier survival curve distribution (Fig. 2F).

### Assessing the clinical value of the prognostic risk model

**Fig. 3:**
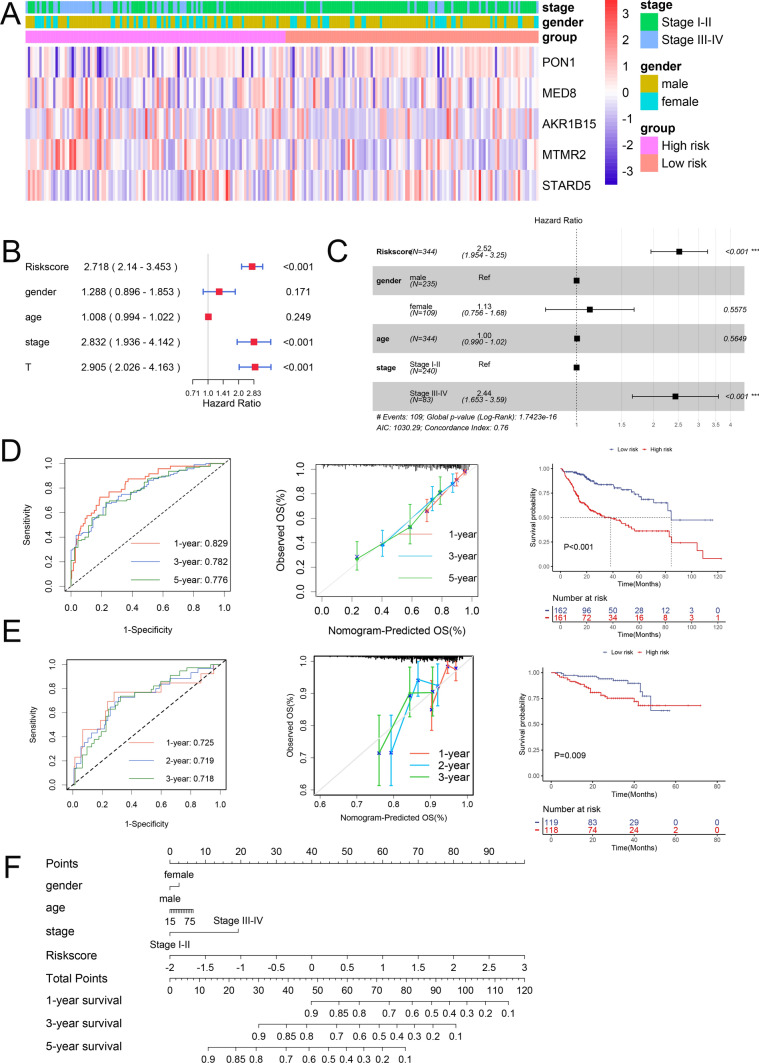
Evaluation of the clinical value of prognostic risk models. **A** Heatmap illustrating the distribution differences among various clinical features between the high- and low-risk score groups. **B** Forest plot depicting Cox regression analysis for univariate clinical variables and **C** multivariate clinical variables. ROC and calibration curves were used to display the survival probabilities of patients with HCC, and the Kaplan–Meier survival curve demonstrated the differences in OS between the high- and low-risk groups in the training cohort **D** and validation cohort **E**. **F **Nomogram for predicting the probabilities of 1-, 3-, and 5-year OS for HCC patients. *ROC* receiver operating characteristic, *HCC* hepatocellular carcinoma, *OS* overall survival

We analysed clinical characteristics, including age, gender, tumour stage, and differences in gene distribution, between the high- and low-risk score groups (Fig. 3A). Following LASSO, univariate and multivariate Cox regression analyses (Fig. 3B, C), we developed a nomogram model for predicting OS (Fig. 3F). We were able to easily estimate the 1-, 3-, and 5-year OS values based on the cumulative scores assigned to each factor via the nomogram. A higher calculated score indicated a less favourable prognosis for the patient. The AUC values of the ROC curve at 1, 3, and 5 years were 0.829, 0.782, and 0.776 (Fig. 3D), respectively, indicating that our model exhibited good sensitivity and specificity in its predictive ability. To evaluate the performance of our nomogram model's predictions, calibration curves were generated that overlapped with the ideal diagonal curve at all time points, including 1 year, 3 years, and 5 years, thus confirming its high predictive accuracy. Furthermore, the validation cohort results confirmed the excellent performance of our model (Fig. 3E). In both the training and validation cohorts, the Kaplan‒Meier survival curve demonstrated a significant reduction in OS within the high-risk score group (P < 0.05).

### Assessing risk models for gene mutations and drug sensitivity

**Fig. 4:**
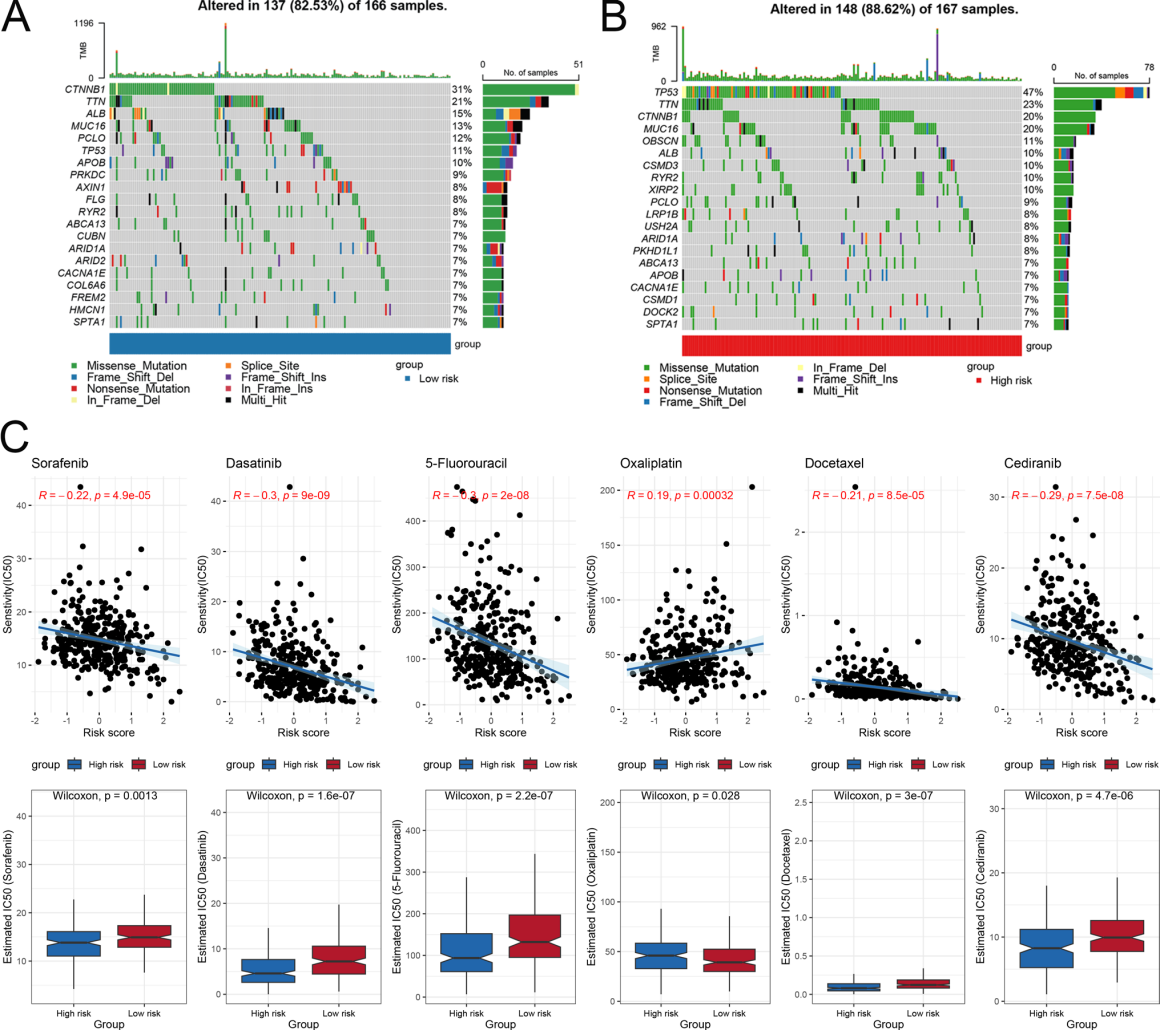
Assessment of the correlations between risk scores, gene mutations, and drug sensitivity. Waterfall plots displaying gene mutation information for the low-risk score group **A** and the high-risk score group **B**. **C** Correlation analysis between the IC_50_ values of six common chemotherapeutic drugs and risk scores, along with comparisons of the IC_50_ values of these drugs in the two risk groups. *IC*_*50*_ half-maximal inhibitory concentration

We calculated the frequencies and distributions of gene mutations in the low-risk score group (Fig. 4A) and the high-risk score group (Fig. 4B). The waterfall plots that were constructed indicated that missense mutations were the predominant mutation type in both groups. The top five mutated genes in the high-risk score group were TP53 (47%), TTN (23%), CTNNB1 (20%), MUC16 (20%), and OBSCN (11%). In contrast, the most commonly mutated genes in the low-risk group were CTNNB1 (31%), TTN (21%), ALB (15%), MUC16 (13%), and PCLO (12%). Notably, TP53 presented a higher mutation frequency in the high-risk score group than did CTNNB1, which had a lower mutation frequency. We subsequently assessed the correlations between risk scores and IC_50_ values of common chemotherapy drugs (|correlation|> 0.15, P < 0.05) (Supplementary File S1) while also analysing differences in drug sensitivity between the two score groups. Our results revealed that sorafenib, dasatinib, 5-fluorouracil, docetaxel, and cediranib demonstrated increased sensitivity in the high-risk group (Fig. 4C), suggesting their potential candidacy for patients with elevated risk scores. Conversely, oxaliplatin had significantly lower IC_50_ values in the low-risk score group than in the high-risk score group, indicating its potential suitability for patients with reduced risk scores.

### Assessing immune cell infiltration and the immune response

**Fig. 5:**
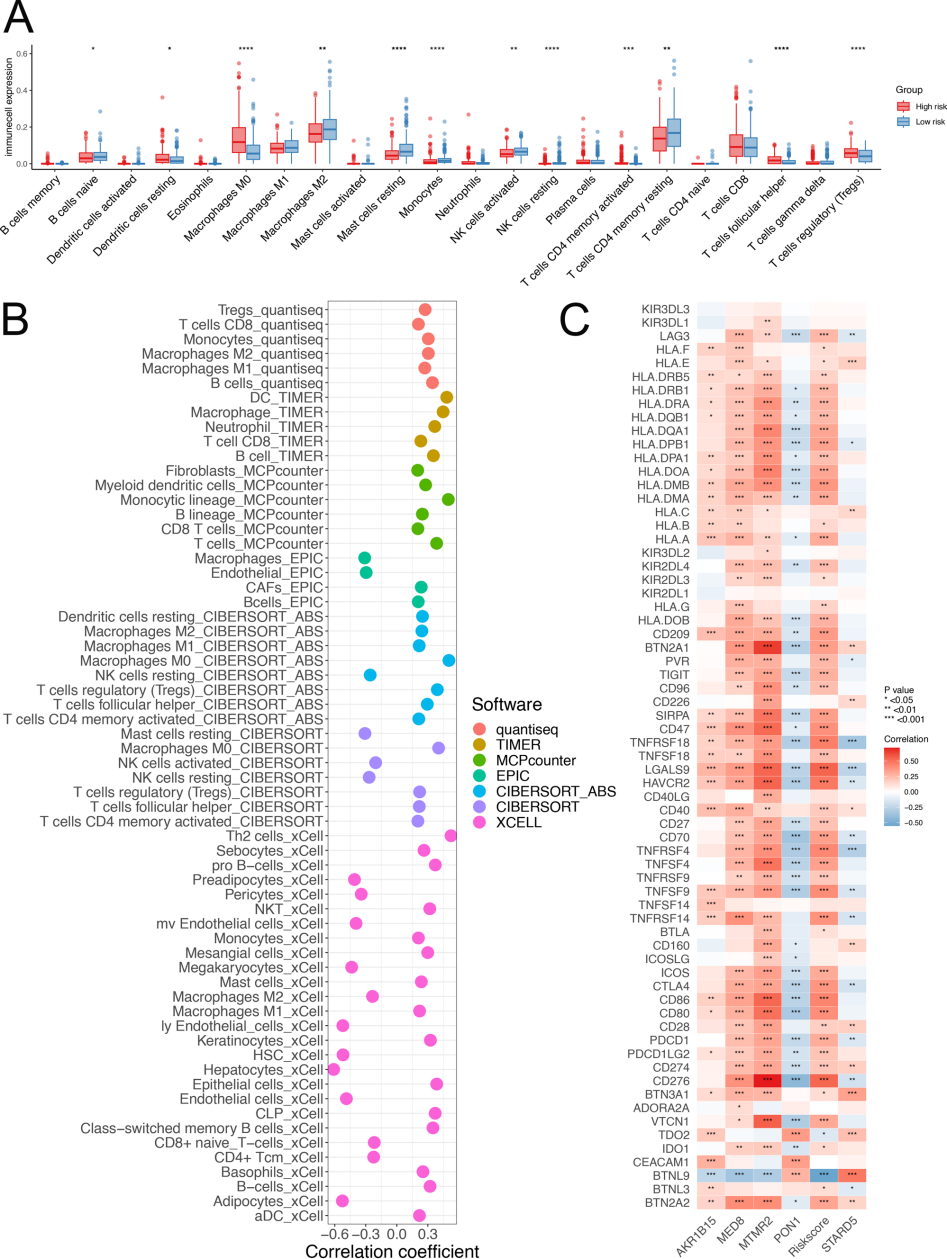
Risk scores were used to assess immune cell infiltration and the immune response. **A** CIBERSORT analysis of differences in immune cell infiltration abundance between the high- and low-risk score groups. **B** Seven methods used to analyse the correlation between immune cell infiltration abundance and the risk score. **C** Correlation analysis of checkpoint gene expression with the risk score and signature genes in the TCGA-LIHC cohort. *TCGA* The Cancer Genome Atlas, *LIHC* liver hepatocellular carcinoma

The TME influences the biological behaviour of tumours. We utilized CIBERSORT to assess the infiltration abundances of 22 immune cell types and compared the disparities between the high- and low-risk score groups in the HCC immune microenvironment. Figure 5A shows that M0 macrophages, resting dendritic cells, follicular helper T cells, and regulatory T cells presented greater infiltration in the high-risk score group. Conversely, naive B cells, M2 macrophages, resting mast cells, and monocytes exhibited increased infiltration in the low-risk group. We subsequently employed the CIBERSORT, MCPCOUNTER, QUANTISEQ, EPIC, TIMER, CIBERSORT-ABS, and XCELL methods to investigate the correlations between the risk score and immune cell infiltration abundance. As depicted in Fig. 5B (|correlation|> 0.2, P < 0.05), most immune cells, including M0 macrophages, CD8(+) T cells, and monocytes, were significantly positively correlated with the risk score. Notably, a significant negative correlation was observed between the risk score and the abundance of recognized antitumour immune cells, such as activated natural killer (NK) cells and resting NK cells. Interestingly, contrasting results emerged when the correlations between the risk score and macrophages were analysed using the TIMER and EPIC methods. Furthermore, we examined the associations between the risk score and the expression of immune checkpoint genes (Fig. 5C). PON1 was significantly negatively correlated with most immune checkpoint genes, whereas the risk score was significantly positively correlated with these genes. Notably, the expression levels of multiple TNF and HLA genes were positively correlated with the risk score.

### scRNA-seq analysis of characteristic genes

**Fig. 6:**
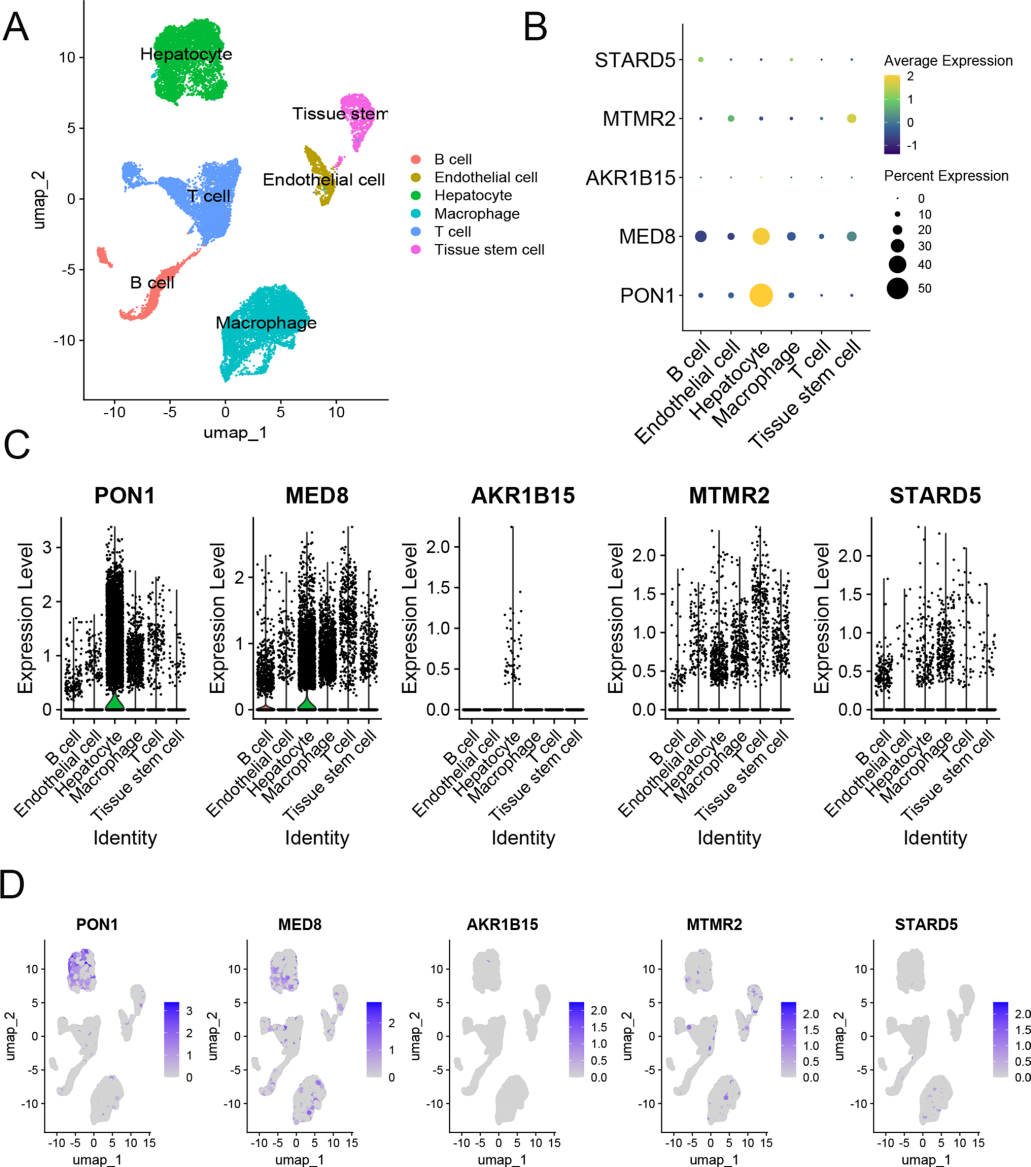
Evaluation of the expression and distribution of five characteristic genes by scRNA-seq. **A** UMAP plot displaying annotations of the dimensionally reduced dataset (GSE149614), with dot plots **B** and violin plots **C** illustrating the expression levels of the five genes in different cell types; **D** UMAP plot of the distribution of the five genes in the single-cell tumour microenvironment. *UMAP* uniform manifold approximation and projection

We selected primary tumours from GSE149614 dataset for reconstruction to explore the distribution and expression of characteristic genes in the TME. We clustered the cells into six subgroups and further annotated them into six cell types: hepatocytes, macrophages, T cells, endothelial cells, tissue stem cells, and B cells (Fig. 6A). The expression and distribution of the five characteristic genes are illustrated in Fig. 6B. The PON1 and MED8 genes were widely expressed and distributed in the TME (Fig. 6C), whereas the MTMR2 and STARD5 genes were moderately expressed and distributed (Fig. 6D). In contrast, AKR1B15 was exclusively expressed and distributed in hepatocyte cells.

### Experimental validation of characteristic genes

**Fig. 7:**
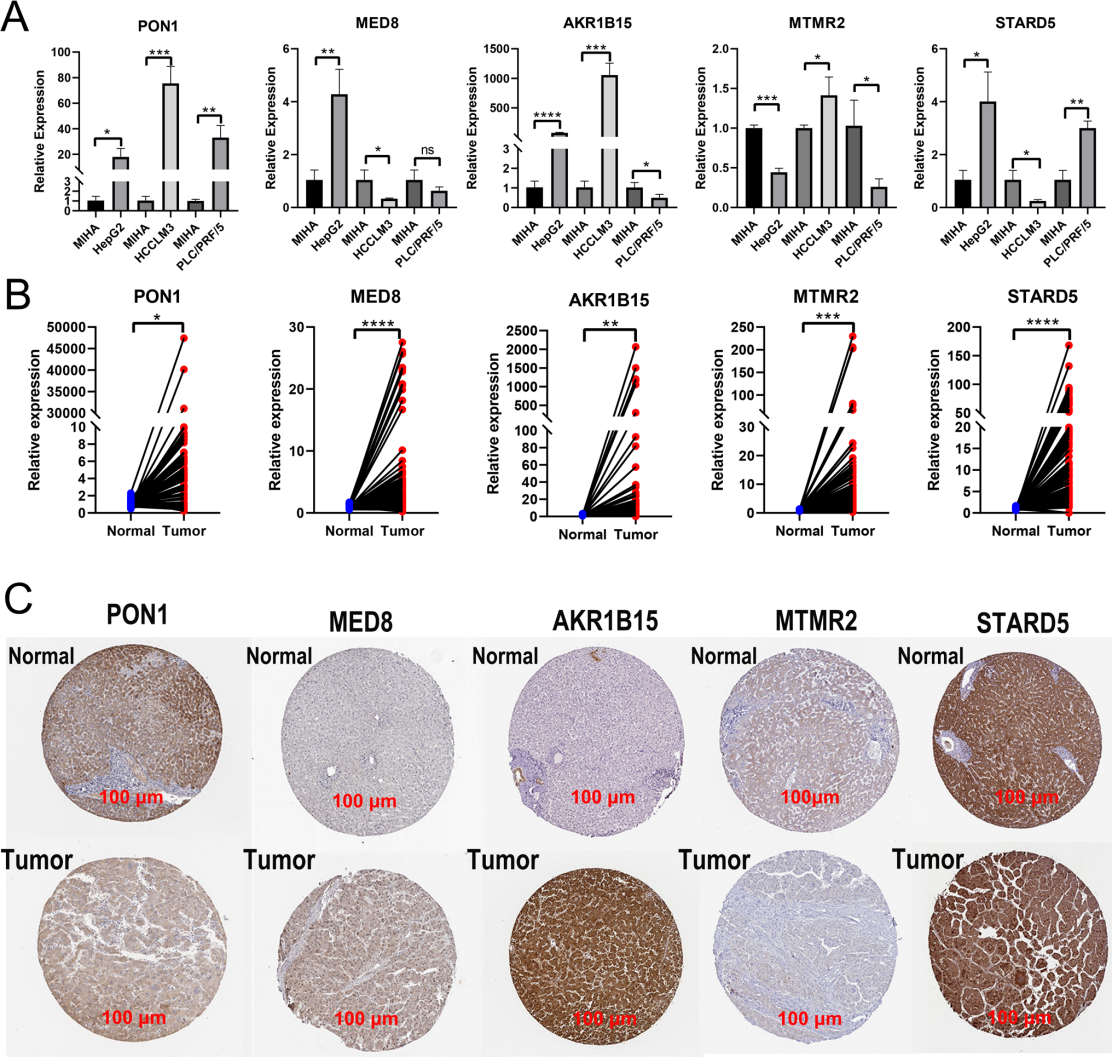
Validation of the expression levels of the five characteristic genes. **A** Expression levels of the five genes in cell lines. **B** Expression levels of the five genes in HCC and adjacent tissues. **C** Immunohistochemical images showing the protein expression of the five genes in the HPA database. (*P < 0.05, **P < 0.01, ***P < 0.001, ****P < 0.0001, ns: not significant). *HCC* hepatocellular carcinoma, *HPA* Human Protein Atlas

We verified the relative expression levels of five characteristic genes in the cell lines. Except for MTMR2, the other genes were highly expressed in HepG2 cells; PON1, AKR1B15, and MTMR2 were highly expressed in HCCLM3 cells, whereas MED8 and STARD5 were expressed at low levels in HCCLM3 cells. In PLC/PRF/5 cells, PON1 and STARD5 were highly expressed (Fig. 7A). qRT‒PCR analysis of 49 pairs of HCC and adjacent tissues from patients revealed that the characteristic genes were highly expressed in HCC tissues and expressed at low levels in adjacent tissues (Fig. 7B). Immunohistochemical results from the Human Protein Atlas (HPA) database confirmed that MED8 and AKR1B15 protein expression levels were relatively high in HCC tissues, whereas PON1 and MTMR2 protein expression levels were lower in HCC tissues than in normal liver tissues (Fig. 7C).

## Discussion

TAMs play crucial roles in regulating tumour proliferation, metastasis, and remodelling of the TME, and are commonly associated with an unfavourable prognosis in patients with cancer [15, 16]. Dysregulation of lipid metabolism-related genes in tumour tissue affects the TME through reprogramming and promotes tumorigenesis and progression. Therefore, characterizing the prognosis and treatment response of patients with HCC by integrating TAMs and lipid metabolism reprogramming is highly important. In this study, we analysed data from the TCGA database to determine whether the abundance of M0 macrophages within tumour tissue impacts the prognosis of patients with HCC. Consequently, we performed a correlation analysis between lipid metabolism genes related to M0 macrophages in HCC. Through LASSO and Cox regression analyses, we developed a prognostic model based on five genes (PON1, MED8, AKR1B15, MTMR2, and STARD5) to reflect chemotherapy drug sensitivity and immune status within the TME. Our findings demonstrate the promising clinical applicability of the risk score for evaluating HCC prognosis as well as for assessing chemotherapy drug sensitivity and immune checkpoint modulation.

Using the CIBERSORT method, we assessed the infiltration of 22 immune cell types in liver cancer tissues. Our findings revealed a significantly poorer prognosis for patients with HCC with high M0 macrophage infiltration, suggesting that targeting the accumulation of M0 macrophages in tumour tissues could be an effective strategy to improve patient outcomes. According to our KEGG pathway enrichment analysis of the 153 lipid-related genes associated with M0 macrophages, particular attention should be given to the glycerophospholipid metabolism pathway. Glycerophospholipid metabolism involves phosphatidylserine (PS), which is present in cellular membranes across various organelles, such as the endoplasmic reticulum, mitochondria, Golgi apparatus, and plasma membrane. PS serves as a potent"eat me"signal for apoptotic phagocytosis and can directly bind to macrophage receptors [17]. Additionally, PS released by apoptotic cells can induce polarization towards M2 macrophages [18]. Furthermore, our GO enrichment analysis identified fatty acid metabolism as the top-ranked biological process. Wu et al. demonstrated that RIPK3 deficiency in TAMs promoted fatty acid metabolism, leading to fatty acid oxidation and activation of the PPAR pathway and thereby facilitating the accumulation of M2 macrophages within the TME [19]. Collectively, these existing studies suggest that reprogramming lipid metabolism may drive M2 macrophage polarization, favouring tumorigenesis. Studies have reported that alpha-fetoprotein can increase the migratory ability of M0 macrophages by activating the PI3 K/AKT signalling pathway, promoting the polarization of M0 macrophages into M2 macrophages [20]. The PI3 K/AKT signalling pathway regulates lipid metabolism in HCC [21], and the subtypes of the PI3 K and AKT genes from the respective families were associated with poor prognosis in patients with HCC [22, 23]. Based on this evidence, we hypothesize that increased infiltration of M0 macrophages within tumour tissue may modulate changes in the TME by reprogramming lipid metabolism-related genes, ultimately promoting the polarization and accumulation of M2 macrophages. Targeting the infiltration of M0 macrophages in the TME holds promise as a strategy to combat HCC. However, research on the specific mechanisms underlying the interaction between M0 macrophages and HCC is limited.

Previous studies have identified the roles of lipid metabolism genes in HCC prognostic models; however, these studies lack a comprehensive understanding of the associations between the model and immune cell populations. The selection of five genes in our prognostic risk model is based on their inherent link with M0 macrophages and their involvement in lipid metabolism. PON1 can attenuate the macrophage inflammatory response [24] and regulate macrophage anti-atherosclerosis activities [25]. The HDL/ApoA1/PON1 complex regulates metabolic reprogramming and macrophage polarization through redox control mechanisms [26]. Wang et al.'s prognostic model explored multiple genes, including PON1, as key regulators of macrophages [27]; nevertheless, experimental verification regarding the regulatory relationships between these genes and macrophages is lacking. MED8 functions as a regulator of polymerase activity and its high expression is associated with poor prognosis [28]. Knockdown experiments demonstrated that reducing MED8 expression weakens HepG2 and Huh7 cell proliferation and migration [29]. Our study revealed significantly increased MED8 gene expression levels in HCC tissues, suggesting that targeting MED8 gene expression may modulate liver cancer development. AKR1B15 belongs to the aldo–keto reductase (AKR) superfamily, is highly expressed in adipose tissue, and is potentially involved in the regulation of steroid metabolism [30]. Yuan et al. [31] showed that AKR1B15 was highly expressed in liver cancer tissues. Its addition to the risk model predicted the prognosis of patients with HCC, which is consistent with our results. Notably, in our scRNA-seq expression analysis, AKR1B15 was expressed exclusively in liver cancer tissues and not in macrophages. MTMR2 has been implicated in the regulation of numerous cellular biological processes, including the cell cycle, the cellular stress response, apoptosis, and signal transduction. It is highly expressed in various cancers, such as HCC, and is associated with a poor prognosis in patients with HCC [32]. Given its regulatory potential in cancer [33–35], MTMR2 is a promising biomarker; however, comprehensive research on the interaction mechanism between MTMR2 and macrophages is lacking. STARD5 is a stress-responsive protein that regulates plasma membrane cholesterol and intracellular cholesterol homeostasis and reduces lipid accumulation in hepatocytes [36]. Liu et al.[37] identified STARD5 as a potential prognostic marker for HCC, with higher expression levels indicating a better prognosis among patients with HCC. Li et al. [38] also reported that STARD5 expression decreased in tumour tissues and that the overexpression of STARD5 inhibited the migration and invasion of HCC cells. In contrast to previous findings, our study demonstrated that Cox regression analysis identified STARD5 as a protective factor for OS (HR = 0.159) and revealed its high expression within liver tumour tissues. STARD5 is present in the cytoplasmic lysate of macrophages and maintains loose binding to the Golgi apparatus [39], and STARD5 gene ablation leads to increased neutral lipid accumulation in macrophages [36]. Our study revealed that STARD5 is expressed in various cells, including macrophages and hepatocytes. However, Rodriguez-Agudo et al.'s [40] Western blot analysis revealed that STARD5 was present in macrophages but not in hepatocytes.

TP53 mutations are frequently observed as genetic alterations in HCC genes, with an average mutation frequency of 30% [41] and a prevalence of approximately 60% in hepatitis B virus (HBV) infected patients with HCC [42]. Our findings demonstrated that the high-risk score group presented the highest frequency of TP53 mutations, reaching 47%, which was significantly greater than the 11% observed in the low-risk score group. Yang et al. reported that prognostic models developed for the HCC population based on the frequency of TP53 mutation had significantly better performance in predicting outcomes [43]. TP53 mutations are considered potential indicators of poor prognosis in patients with cancer [44], and our study revealed that patients in the high-risk score group with high frequencies of TP53 mutations experienced unfavourable outcomes. Additionally, our investigation identified CTNNB1 as a distinctive feature specific to the low-risk group. Mechanistically, Senni et al. showed that CTNNB1, which encodes β-catenin in the Wnt pathway, can block the development of HCC by targeting the transcription factor Pparα, thereby blocking the metabolic reprogramming of fatty acid oxidation activated by β-catenin [45]. We compared patient sensitivity to commonly used chemotherapy drugs between the high- and low-risk score groups and found that patients in the high-risk score group exhibited greater sensitivity to most therapeutic agents (such as sorafenib, dasatinib, and 5-fluorouracil), suggesting that these drugs may represent potential candidates for treating patients with HCC with poor prognoses.

We investigated the potential role of risk score and immune cells. The high-risk group presented a greater abundance of M0 macrophages, whereas the low-risk group presented a greater abundance of M2 macrophages, suggesting that the unfavourable prognosis associated with high risk scores may be attributed to increased infiltration of M0 macrophages and decreased infiltration of M2 macrophages. Additionally, we observed distinct differences in various T-cell subtypes between the high- and low-risk groups, indicating a potential regulatory relationship between T cells and lipid metabolism reprogramming. Notably, regulatory T cells within the tumour microenvironment rely heavily on the mitochondrial oxidative metabolism of lipids rather than glucose [46]. Immune checkpoint genes have emerged as promising targets for cancer therapy, and the blockade of immune checkpoints represents a potential strategy for cancer immunotherapy [47]. HLA genes play crucial roles in identifying tissue-specific antigens and presenting them to immune cells, thereby influencing the efficacy of immunotherapy. Our risk scores demonstrated positive correlations with multiple HLA genes, suggesting their potential utility as biomarkers. Furthermore, our findings highlight associations between risk scores and several tumour necrosis factor superfamily ligands and receptor superfamilies that are pivotal for modulating immune system function. Pichler et al. revealed that TNFRSF9 stimulation can enhance anti-PD1 effectiveness, thus underscoring its potential as an immunotherapeutic target [48].

scRNA-seq is a powerful tool for elucidating tumour heterogeneity at the single-cell level in cancer [49]. We analysed the expression profiles of five signature genes in individual cells and revealed high MED8 expression across various cell types, particularly macrophages, T cells, and hepatocytes. These gene signatures serve as valuable references for characterizing infiltrating immune cells within the TME. For example, the expression levels of characteristic genes were low or absent in infiltrated B cells, suggesting that the regulatory effect of characteristic genes on lipid metabolism is probably not directly initiated by B cells. However, our study has certain limitations. First, potential selection bias may exist due to our reliance on publicly available datasets. Thus, further validation with real-world data is needed to confirm the robustness of our prognostic model. Second, additional in vivo and in vitro experiments are necessary to elucidate the regulatory mechanisms underlying M0 macrophages and lipid metabolism genes as potential targets for immunotherapy.

## Conclusions

In summary, we identified five lipid metabolism genes associated with M0 macrophages as prognostic markers, leading to the construction of a robust prognostic model with potential clinical applicability. This model offers significant value for survival prediction and guiding immunotherapy in patients with HCC. Furthermore, our study provides deeper insights into the functional role of M0 macrophages in HCC, facilitating the development of more effective liver cancer treatments.

## Supplementary Information

Additional file 1

## Data Availability

The datasets analysed during the current study are available in the following repositories: GEO (https://www.ncbi.nlm.nih.gov/geo/), ICGC (https://dcc.icgc.org/), TCGA (https://www.cancer.gov/ccg/), and MSigDB (http://www.broad.mit.edu/gsea/msigdb/).
