# Supplementary material for: Identification of M0 macrophage associated lipid metabolism genes for prognostic and immunotherapeutic response prediction in hepatocellular carcinoma
Source: Discov Oncol. 2025 May 16;16:781. doi: 10.1007/s12672-025-02620-1 (PMC12084471; doi:10.1007/s12672-025-02620-1)
Supplement: Supplementary file 1 — Additional file 1 [file 12672_2025_2620_MOESM1_ESM.docx]

1. Primers for qRT–PCR

| Transcript | Forward | Reverse |
| --- | --- | --- |
| PON1 | TTCAGGAACCACCAGTCTTCTTACC | TCTCCAAGTCTTCAGAGCCAGTTTC |
| MED8 | AACCCTACAGACACTAATGCCTTGG | CCACTGCTGCCTGAAGGTCTC |
| AKR1B15 | TCCAGCATCTCTTCTCGGCAAAG | TGGATCTTCTCTTGGATGGCTTCTC |
| MTMR2 | TCCTTAGCCTCCTTCGACCT | TCCAGGTGCACAAGACAGAC |
| STARD5 | GGAGGTGTGGGACTGTGTGAAG | AGCGGAGGGAGTGGAGGTTC |

2. A total of 106 chemotherapy drugs are associated with this study

|  | Chemotherapy drugs | Correlation (TCGA) | p value |
| --- | --- | --- | --- |
| 1 | Vinblastine_1004 | -0.445184211 | 0 |
| 2 | Docetaxel_1007 | -0.384970495 | 1.89E-13 |
| 3 | Navitoclax_1011 | -0.283902982 | 1.01E-07 |
| 4 | Vorinostat_1012 | -0.212262384 | 7.71E-05 |
| 5 | Nilotinib_1013 | -0.214760917 | 6.32E-05 |
| 6 | Axitinib_1021 | -0.415692768 | 0 |
| 7 | AZD7762_1022 | -0.348745321 | 4.07E-11 |
| 8 | Afatinib_1032 | -0.23982625 | 7.54E-06 |
| 9 | Staurosporine_1034 | -0.327928566 | 6.14E-10 |
| 10 | NU7441_1038 | 0.229451476 | 1.87E-05 |
| 11 | Doramapimod_1042 | 0.50509877 | 0 |
| 12 | Wee1 Inhibitor_1046 | -0.416479321 | 0 |
| 13 | Nutlin-3a (-)_1047 | 0.287142274 | 7.11E-08 |
| 14 | ZM447439_1050 | -0.180727685 | 0.000785872 |
| 15 | Palbociclib_1054 | -0.223482889 | 3.10E-05 |
| 16 | Pictilisib_1058 | -0.263718742 | 7.98E-07 |
| 17 | AZD8055_1059 | -0.233719087 | 1.29E-05 |
| 18 | 5-Fluorouracil_1073 | -0.326703684 | 7.15E-10 |
| 19 | Dasatinib_1079 | -0.34974301 | 3.56E-11 |
| 20 | Paclitaxel_1080 | -0.495009772 | 0 |
| 21 | Rapamycin_1084 | -0.366563656 | 3.31E-12 |
| 22 | Sorafenib_1085 | -0.189773794 | 0.000418539 |
| 23 | Oxaliplatin_1089 | 0.169176798 | 0.001685797 |
| 24 | BMS-536924_1091 | -0.255037213 | 1.85E-06 |
| 25 | GSK1904529A_1093 | -0.188298225 | 0.000464747 |
| 26 | Tozasertib_1096 | -0.373755045 | 1.13E-12 |
| 27 | PF-4708671_1129 | -0.383452997 | 2.43E-13 |
| 28 | MK-1775_1179 | -0.541361413 | 0 |
| 29 | Dinaciclib_1180 | -0.314808438 | 3.06E-09 |
| 30 | Bortezomib_1191 | -0.391199879 | 6.36E-14 |
| 31 | GSK269962A_1192 | -0.240586635 | 7.05E-06 |
| 32 | SB505124_1194 | 0.51339966 | 0 |
| 33 | Fulvestrant_1200 | -0.260005139 | 1.15E-06 |
| 34 | YK-4-279_1239 | -0.424763261 | 0 |
| 35 | Daporinad_1248 | -0.301357035 | 1.47E-08 |
| 36 | BMS-345541_1249 | -0.283620477 | 1.04E-07 |
| 37 | AZ960_1250 | -0.22787599 | 2.14E-05 |
| 38 | Dabrafenib_1373 | -0.1521355 | 0.004783331 |
| 39 | Temozolomide_1375 | -0.328556917 | 5.67E-10 |
| 40 | IAP_5620_1428 | 0.306586055 | 8.06E-09 |
| 41 | AZD2014_1441 | 0.265546103 | 6.66E-07 |
| 42 | AZD1208_1449 | 0.271052869 | 3.83E-07 |
| 43 | Cyclophosphamide_1512 | -0.255593897 | 1.76E-06 |
| 44 | Pevonedistat_1529 | -0.396058668 | 2.51E-14 |
| 45 | Sapitinib_1549 | -0.188324097 | 0.000463897 |
| 46 | LCL161_1557 | 0.170514088 | 0.001546862 |
| 47 | Lapatinib_1558 | -0.363345181 | 5.29E-12 |
| 48 | Luminespib_1559 | -0.173406345 | 0.001281603 |
| 49 | Alpelisib_1560 | -0.282979934 | 1.11E-07 |
| 50 | Taselisib_1561 | -0.225830059 | 2.54E-05 |
| 51 | SCH772984_1564 | -0.16465761 | 0.002244272 |
| 52 | Leflunomide_1578 | -0.286190084 | 7.87E-08 |
| 53 | Entinostat_1593 | 0.190425042 | 0.000399537 |
| 54 | LGK974_1598 | -0.286361371 | 7.73E-08 |
| 55 | VE-822_1613 | -0.164756338 | 0.002230452 |
| 56 | CZC24832_1615 | -0.155797657 | 0.003854633 |
| 57 | PFI3_1620 | -0.161741563 | 0.002689437 |
| 58 | Wnt-C59_1622 | -0.327152421 | 6.76E-10 |
| 59 | RVX-208_1625 | -0.19243737 | 0.000345778 |
| 60 | ML323_1629 | -0.526038042 | 0 |
| 61 | Entospletinib_1630 | -0.157253896 | 0.003533153 |
| 62 | Ribociclib_1632 | -0.164714706 | 0.00223627 |
| 63 | AGI-6780_1634 | -0.189883822 | 0.000415271 |
| 64 | Eg5_9814_1712 | -0.155495228 | 0.003924631 |
| 65 | ERK_6604_1714 | -0.246776172 | 4.01E-06 |
| 66 | JAK1_8709_1718 | 0.331278481 | 4.02E-10 |
| 67 | PAK_5339_1730 | -0.293091235 | 3.71E-08 |
| 68 | ULK1_4989_1733 | -0.393176225 | 4.41E-14 |
| 69 | Selumetinib_1736 | 0.257631502 | 1.44E-06 |
| 70 | Ibrutinib_1799 | -0.241891511 | 6.27E-06 |
| 71 | Oxaliplatin_1806 | 0.216875839 | 5.33E-05 |
| 72 | Teniposide_1809 | -0.203861279 | 0.000148147 |
| 73 | Mitoxantrone_1810 | 0.150660526 | 0.005211209 |
| 74 | Fulvestrant_1816 | -0.259060977 | 1.26E-06 |
| 75 | Vincristine_1818 | -0.359395463 | 9.31E-12 |
| 76 | Docetaxel_1819 | -0.343782451 | 7.93E-11 |
| 77 | Podophyllotoxin bromide_1825 | -0.225583536 | 2.60E-05 |
| 78 | Gallibiscoquinazole_1830 | -0.304640338 | 1.01E-08 |
| 79 | MG-132_1862 | -0.368195643 | 2.60E-12 |
| 80 | BDP-00009066_1866 | -0.344782816 | 6.94E-11 |
| 81 | Buparlisib_1873 | -0.2989605 | 1.93E-08 |
| 82 | AZD6738_1917 | -0.442950935 | 0 |
| 83 | Osimertinib_1919 | -0.293613424 | 3.50E-08 |
| 84 | Cediranib_1922 | -0.279769488 | 1.56E-07 |
| 85 | Ipatasertib_1924 | -0.167532024 | 0.001872336 |
| 86 | GDC0810_1925 | -0.454756968 | 0 |
| 87 | GNE-317_1926 | -0.178379028 | 0.000921226 |
| 88 | GSK2578215A_1927 | -0.18597306 | 0.00054728 |
| 89 | I-BRD9_1928 | -0.342788628 | 9.05E-11 |
| 90 | Telomerase Inhibitor IX_1930 | -0.33908008 | 1.47E-10 |
| 91 | P22077_1933 | -0.156760851 | 0.003639169 |
| 92 | UMI-77_1939 | -0.247748286 | 3.67E-06 |
| 93 | WIKI4_1940 | -0.36904524 | 2.30E-12 |
| 94 | Sepantronium bromide_1941 | -0.48559492 | 0 |
| 95 | MIM1_1996 | -0.323191998 | 1.11E-09 |
| 96 | WEHI-539_1997 | -0.305124463 | 9.54E-09 |
| 97 | BPD-00008900_1998 | -0.412573912 | 0 |
| 98 | MK-8776_2046 | -0.206863266 | 0.000117653 |
| 99 | Ulixertinib_2047 | -0.343599566 | 8.12E-11 |
| 100 | Vinorelbine_2048 | -0.404238824 | 3.52E-15 |
| 101 | VX-11e_2096 | -0.198367002 | 0.000223999 |
| 102 | Uprosertib_2106 | 0.183115596 | 0.000667314 |
| 103 | LJI308_2107 | 0.208657322 | 0.000102356 |
| 104 | GSK591_2110 | -0.164110145 | 0.002322335 |
| 105 | VE821_2111 | -0.392472044 | 5.03E-14 |
| 106 | AZD6482_2169 | 0.240534892 | 7.08E-06 |
| 107 | BMS-754807_2171 | -0.151615988 | 0.004930281 |
